# Characterization of an Open-Access Medical News Platform’s Readership During the COVID-19 Pandemic: Retrospective Observational Study

**DOI:** 10.2196/26666

**Published:** 2021-05-25

**Authors:** Alex K Chan, Constance Wu, Andrew Cheung, Marc D Succi

**Affiliations:** 1 Michael G. DeGroote School of Medicine McMaster University Hamilton, ON Canada; 2 Medically Engineered Solutions in Healthcare Incubator, Innovation in Operations Research Center Massachusetts General Hospital Boston, MA United States; 3 Harvard Medical School Boston, MA United States; 4 Department of Medicine Faculty of Health Sciences McMaster University Hamilton, ON Canada; 5 Department of Radiology Massachusetts General Hospital Boston, MA United States

**Keywords:** COVID-19, internet, medical news, text summaries, readership trends, news, media, open access, literature, web-based health information, survey, cross-sectional, trend

## Abstract

**Background:**

There are many alternatives to direct journal access, such as podcasts, blogs, and news sites, that allow physicians and the general public to stay up to date with medical literature. However, there is a scarcity of literature that investigates the readership characteristics of open-access medical news sites and how these characteristics may have shifted during the COVID-19 pandemic.

**Objective:**

This study aimed to assess readership and survey data to characterize open-access medical news readership trends related to the COVID-19 pandemic and overall readership trends regarding pandemic-related information delivery.

**Methods:**

Anonymous, aggregate readership data were obtained from 2 Minute Medicine, an open-access, physician-run medical news organization that has published over 8000 original, physician-written texts and visual summaries of new medical research since 2013. In this retrospective observational study, the average number of article views, number of actions (defined as the sum of the number of views, shares, and outbound link clicks), read times, and bounce rates (probability of leaving a page in <30 s) were compared between COVID-19 articles published from January 1 to May 31, 2020 (n=40) and non–COVID-19 articles (n=145) published in the same time period. A voluntary survey was also sent to subscribed 2 Minute Medicine readers to further characterize readership demographics and preferences, which were scored on a Likert scale.

**Results:**

COVID-19 articles had a significantly higher median number of views than non–COVID-19 articles (296 vs 110; *U*=748.5; *P*<.001). There were no significant differences in average read times (*P*=.12) or bounce rates (*P*=.12). Non–COVID-19 articles had a higher median number of actions than COVID-19 articles (2.9 vs 2.5; *U*=2070.5; *P*=.02). On a Likert scale of 1 (strongly disagree) to 5 (strongly agree), our survey data revealed that 65.5% (78/119) of readers agreed or strongly agreed that they preferred staying up to date with emerging literature about COVID-19 by using sources such as 2 Minute Medicine instead of journals. A greater proportion of survey respondents also indicated that open-access news sources were one of their primary sources for staying informed (86/120, 71.7%) compared to the proportion who preferred direct journal article access (61/120, 50.8%). The proportion of readers indicating they were reading one or less full-length medical studies a month were lower following introduction to 2 Minute Medicine compared to prior (21/120, 17.5% vs 38/120, 31.6%; *P*=.005).

**Conclusions:**

The readership significantly increased for one open-access medical literature platform during the pandemic. This reinforces the idea that open-access, physician-written sources of medical news represent an important alternative to direct journal access for readers who want to stay up to date with medical literature.

## Introduction

On March 11, 2020, COVID-19 was declared a pandemic by the World Health Organization roughly 11 weeks after the report of the first detected COVID-19 case [1]. With its high transmissibility and global impact, COVID-19 has attracted tremendous interest from researchers, clinicians, and the general population worldwide. Within the first 3 months of the disease’s discovery, bibliometric analyses indicated that there was already a greater number of peer-reviewed COVID-19 studies published than the combined number of articles on SARS (severe acute respiratory syndrome) and MERS (Middle East respiratory syndrome) published during these diseases’ first year of discovery [2,3]. This has also resulted in significant interest from the mass media, as a substantial increase in COVID-19–related news was observed in the early months of 2020 [4]. Prior literature has found that overall news consumption increases significantly during a national crisis, and this appears to be the case for the COVID-19 outbreak, as there is evidence suggesting that information-seeking behavior increased following the declaration of the pandemic [5-7].

Compared to the past, many alternatives to direct journal access and mainstream media now exist for clinicians and the general public who want to stay informed about medical news and research, including, but not limited to, social media, blogs, journal newsletters, open-access medical news sites, and podcasts. Although there is evidence that suggests that differences in the use of these alternative sources can have subsequent downstream effects on consumers’ health behaviors, there is little data on the characterization of the consumption of these sources both prior to and following the start of the pandemic [8]. Specifically, there is a notable scarcity of investigations examining the readership trends and characteristics of open-access medical news organizations.

To further characterize the consumption of medical literature during the COVID-19 pandemic, this study sought to investigate readership trends for one physician-run, open-access medical news organization and readership preferences regarding the delivery of COVID-19–related research and information. 

## Methods

### Readership Data

Aggregate, anonymous data for this retrospective observational study was obtained from 2 Minute Medicine Inc [9]. 2 Minute Medicine is a free, open-access medical news organization that publishes daily, physician-written texts and visual summaries of new medical research. It has published over 8000 summaries since 2013. Articles published on the website from January 1 to May 31, 2020, were included in our analysis. In that time span, 40 articles were published about COVID-19–related research, and 145 articles were published about non–COVID-19 medical research. Overall daily website traffic, average view times, the average number of actions, and average bounce rates were longitudinally characterized for the study period.

### Web-Based Survey Data

A web-based survey was sent to 4221 readers who opted into the website’s free, daily electronic mailing list. Nonidentifying demographic data, including age, sex, respondents’ level of education, and respondents’ field of work or study, were gathered. Survey items were created with the intention of directly addressing identified gaps in literature regarding respondents’ perspectives and the relative use of alternative sources, as secondary outcome validation was beyond the scope of this study. Descriptive data were gathered on readers’ behaviors and preferences via Likert scales that ranged from 1 (strongly disagree) to 5 (strongly agree). The key statements used in the study included items such as “I prefer using open access medical news sites such as www.2minutemedicine.com to stay up to date with research related to COVID-19.” Users were also asked to indicate all of the primary sources that they were using to stay informed about COVID-19–related literature, including, but not limited to, direct journal access, journal newsletters, mainstream media, social media, and open-access medical news sites such as 2 Minute Medicine. Other readership trends were also briefly investigated with the survey, as users indicated their frequency of reading full-length original journal articles both prior to and following their introduction to 2 Minute Medicine.

### Ethics

This study was compliant with the Health Insurance Portability and Accountability Act and exempt from review by the institutional review board.

### Statistical Analysis

All statistical analyses were performed using SPSS, version 26 (IBM Corporation). The visual inspection of a histogram, normal quantile-quantile plot, and box plot and the Kolmogorov-Smirnov test were completed to determine if the data followed a normal distribution [10]. If the data were not normally distributed, the Mann-Whitney *U* test was used to compare the average number of views (over the first 2 weeks following publication on the website), number of actions (defined as the sum of the number of views, shares, and outbound link clicks), read times (in seconds), and bounce rates (defined as the probability of readers leaving the webpage in <30 s) for each article [11]. In this study, actions, read times, and bounce rates were used as measures of reader engagement.

## Results

A total of 121 responses were obtained over a 7-week period (response rate: 121/4421, 2.7%). Demographic characteristics of respondents are summarized in Table 1. The majority of respondents reported that they worked in the health care industry (114/121, 94.2%). These respondents included undergraduate students, graduate students, medical students, medical residents, and licensed physicians, and they made up the majority of the respondents.

**Table 1:** Demographic characteristics of respondents.

Characteristics			Value, n (%)
**Sex**			
	Male	66 (55)	
	Female	54 (45)	
**Age group (years)**			
	<20	1 (0.8)	
	20-29	43 (35.8)	
	30-39	37 (30.8)	
	40-49	6 (5)	
	50-59	7 (5.8)	
	60-69	10 (8.3)	
	>69	9 (7.5)	
	Did not indicate	6 (5)	
**Education**			
	Less than or equal to high school graduate	3 (2.5)	
	Some college or university education	13 (10.8)	
	Undergraduate degree	18 (15)	
	Master’s degree	24 (20)	
	Doctoral or professional degree	62 (51.6)	
**Field of work or study**			
	Health care worker	116 (95.9)	
	Other	5 (4.1)	
	Licensed physician	26 (21.4)	
	Licensed allied health professional	13 (10.7)	
	Licensed pharmacist	7 (5.7)	
	Medical or surgical fellow	6 (4.9)	
	Medical or surgical resident	34 (28.1)	
	Pharmacy resident	1 (0.8)	
	Medical student	6 (4.9)	
	Graduate student	10 (8.2)	
	Undergraduate student	9 (7.3)	
	Pharmacy student	2 (1.7)	
	Unspecified or other	6 (4.9)	

Over the study period, COVID-19 articles had a total of 68,129 views (mean 1792, SD 6491), while articles not related to the pandemic accrued 19,650 views (mean 137, SD 122). Our analysis via the Mann-Whitney *U* test revealed that COVID-19 articles published within the observed time frame had a significantly higher median number of views than non–COVID-19 articles (296 vs 110; *U*=748.5; *P*<.001). There was no difference in average view times or bounce rates between the two types of articles. Non–COVID-19 articles had a significantly higher median number of actions than COVID-19 articles (2.9 vs 2.5; *U*=2070.5; *P*=.02).

**Figure 1:**
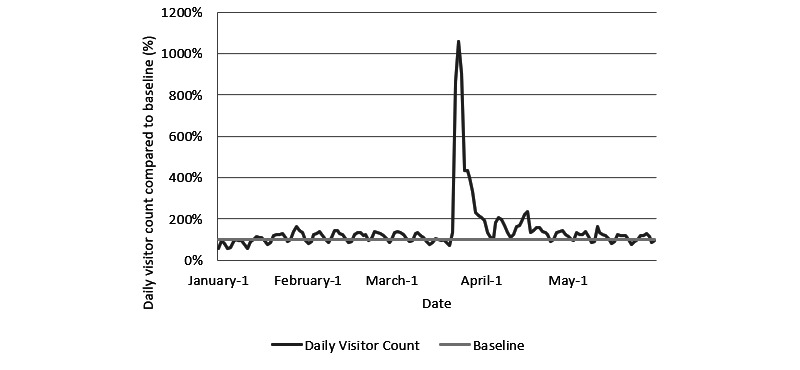
Daily visitor traffic from January 1 to May 30, 2020 compared to the baseline average.

**Figure 2:**
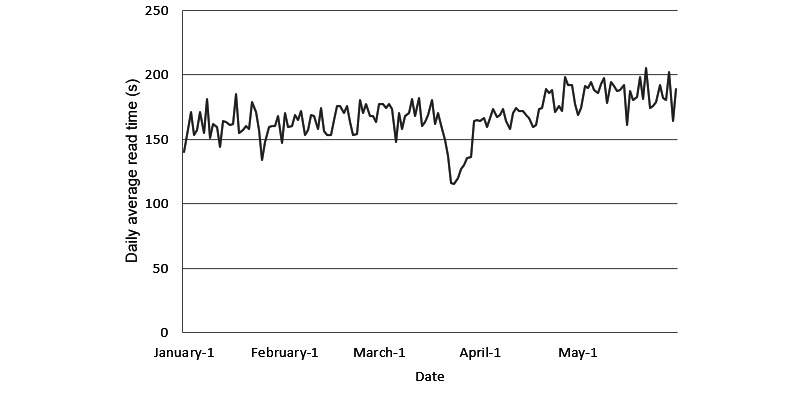
Daily average read times for articles from January 1 to May 30, 2020.

The number of daily visitors over the study period are displayed in Figure 1. The average mean daily visitor count was 1724 (SD 1549). Daily visitor counts were relatively stable from January 1 to February 29, 2020 (mean 1298, SD 292), but a significant peak in daily readership was observed in the month of March (mean 2816, SD 3134), and a peak visitor count of 12,806 daily readers was observed on March 24. The mean daily visitor count from April 1 to May 31 was 1586 (SD 437). The average visit time per page over the study period was 168 s (SD 16 s), and this is shown in Figure 2. The lowest average daily read times were found on March 23 (116 s), March 24 (115 s), and March 25 (120 s).

In terms of survey responses, on a Likert scale of 1 (strongly disagree) to 5 (strongly agree), data from 119 responses (2 respondents did not answer) revealed that 65.5% (78/119) of readers agreed or strongly agreed that they preferred staying up to date with emerging literature about COVID-19 by using sources such as 2 Minute Medicine instead of journals, as shown in Table 2.

When respondents were asked to indicate all of the sources they used to stay up to date with COVID-19 literature, a greater proportion of survey takers indicated that open-access news sources were one of their primary means of staying informed (86/120, 71.7%) compared to the proportion who preferred direct journal article access (61/120, 50.8%), as shown in Table 3. Journal newsletter updates (70/120, 58.3%), social media sources (53/120, 44.2%), and mainstream news media sources (46/120, 38.3%) were other recognized sources of information. The following five other sources were also recognized as sources of information: work or school announcements, podcasts, institutional newsletters, and search engine notifications. In terms of the impact of 2 Minute Medicine on information consumption, a Fisher exact test demonstrated that the proportion of readers who indicated that they were reading 1 or fewer full-length medical studies per month decreased after their introduction to 2 Minute Medicine (21/120, 17.5% vs 38/120, 31.6%; *P*=.005).

**Table 2:** Likert scale data for the question “I prefer staying up to date with emerging research surrounding COVID-19 by using sources such as 2 Minute Medicine versus reading the articles themselves.”

Selection	Responses, n (%)
Strongly Disagree	3 (2.5)
Disagree	10 (8.4)
Neutral	28 (23.5)
Agree	39 (32.8)
Strongly Agree	39 (32.8)

**Table 3:** Sources identified by readers as their primary means for staying informed with COVID-19–related research.

Source	Responses, n (%)
Open-access medical news sites	86 (71.7)
Journal newsletters	70 (58.3)
Direct journal access	61 (50.8)
Social media	53 (44.2)
Mainstream media	46 (38.3)
Research newsletters	20 (16.7)
Research aggregator services	16 (13.3)
Other sources	5 (4.2)

## Discussion

### Principal Results

The results in this study show that average view counts for COVID-19–related articles were significantly higher than those of articles that covered other medical news during the pandemic (*P*<.001). Although this suggests people’s overall interest in COVID-19–related news was greater than their interest in other medical news, there were no associated findings for measures of audience engagement, including average view times or bounce rates. Interestingly however, the average number of actions, which consisted of the number of views, shares, and outbound link clicks per article, were found to be significantly higher for non–COVID-19 medical news articles (*P*=.02), which had an average of nearly 3 actions. We hypothesized that this was due to these articles tending to cover subspecialties or other niche areas of medicine, thereby attracting readers that may be more heavily involved in these fields and have a higher probability of reading primary source articles.

Longitudinal trends of daily visitor counts indicated relatively stable overall site traffic for the first 3 months of 2020. A large spike in daily readership was observed between March 23 to March 26. This was followed by variable daily site traffic before it returned to relative stability in the third and fourth weeks of April. Interestingly, while prior literature has documented increased information-seeking behavior in the first 1 or 2 days following local announcements of outbreaks, this large spike in site traffic appeared to be primarily attributable to a site that published summaries covering research on hydroxychloroquine and its potential therapeutic application for treating COVID-19 [7]. Daily visitor counts dropped in the following weeks but remained above the baseline average. This is possibly indicative of readership retention. Furthermore, while average read times remained fairly stable with an SD of less than 10% over the study period, a substantial decrease in average read times coincided with this peak in visitor count; the decreased average read time was 30.3%. Although this was not confirmed in our site analysis, we believed that this decrease in average read time may be reflective of the site’s larger reach to the general public compared to the normal demographic of the site’s readers—health care professionals. Overall, our results indicate that secondary sources such as medical news sites may have readership data that are more heavily influenced by the gravity and popularity of individual research articles than by other potential factors, such as infection count or other trends related to the pandemic. However, this will need to be further validated in future studies. To our knowledge, these findings represent the first characterization of open-access medical news readership trends during an international health crisis.

In our review of the survey results, the survey data collected appeared to corroborate the findings of the site data comparisons. Unsurprisingly, the majority of respondents (114/121, 94.2%) to the survey, who were subscribers of 2 Minute Medicine, were primarily composed of those directly involved in health care. The survey results indicated that users preferred open-access medical news sources such as 2 Minute Medicine as their primary source of information about pandemic-related research. This is based on both the overall agreement responses on the Likert scale and the fact that a greater proportion of respondents indicated that such sources were their primary news source compared to the proportion who used journals, journal newsletters, and mainstream media as their primary news source. The transition to using sources such as open-access new sources and newsletters may be increasing in prevalence due to the explosive growth in the volume of scientific literature being published per year [11]. The use of these alternative sources may provide readers with a means to sort through such literature and identify research with the highest impact to guide their reading. Interestingly, the use of 2 Minute Medicine appeared to influence the respondents’ overall consumption of medical literature, as readers indicated that they read a greater number of articles via direct journal access following their introduction to the website. In terms of the demographic data acquired in the study, it is of note that despite people’s increased interest in 2 Minute Medicine and its articles covering COVID-19–related research, the vast majority of subscribers to the website continue to be composed of respondents who are directly involved in the health care field. As such, further investigations may be needed to characterize the readership trends and preferences of the general public.

To our knowledge, this is the first study that describes open-access medical news readership trends and preferences for sources alternative to journals during the COVID-19 pandemic. During an outbreak in which an abundance of research is being published while there is high global interest in the pandemic, having a better understanding of how information is consumed by both health care workers and the general public may provide a better understanding of the behaviors associated with the pandemic. Prior literature has demonstrated that different sources of information are at variable risk of misinformation, which may have subsequent impacts on consumer behavior [12-16]. For example, a prior study conducted by Allington et al [8] found a positive correlation between the use of social media and the frequency of COVID-19–related conspiracy theories and a negative relationship between COVID-19–related health-protective behaviors and the use of social media.

### Limitations

In terms of the limitations of this study, as the survey was only sent to subscribers of 2 Minute Medicine—the vast majority of whom are in the field of health care—the generalizability of our findings to the general public are unknown. The sample used for the survey also invariably skewed data to favor preferences toward open-access medical sites. This was a result of sampling bias, as respondents were active subscribers of the organization. Further studies should more broadly survey health care professionals to repeat our findings. It is also recognized that the survey used in this study lacks any validity evidence, and the wording of questions may have been framed positively toward 2 Minute Medicine, which may have introduced bias in the collection of results. Additionally, although the demographics of survey respondents were gathered, individual demographic data on site visitors were not obtained for the purposes of this study, which led to a lack of understanding of the visitors to the site itself. Finally, as survey data on the frequency of reading full-length original studies were not gathered prospectively, this study can only provide limited information regarding the interaction between open-access medical news consumption and original research consumption.

### Conclusions

The results in this study indicate that readership on an open-access medical news site significantly increased as a result of the COVID-19 pandemic. Overall site traffic remained relatively stable in the first 2 months of the study period, and a substantial spike in traffic occurred in mid-March 2020. Based on the comparisons between article view counts and survey data, it appears as though open-access medical news sites may represent an important source for physicians and other health care workers who want to stay up to date with relevant COVID-19 literature. The introduction to such sites may also have subsequent impacts on overall medical literature consumption and increase the frequency of direct journal access.

## Abbreviations

MERS: Middle East respiratory syndrome
SARS: severe acute respiratory syndrome
